# Comparison of univariate and multivariate linkage analysis of traits related to hypertension

**DOI:** 10.1186/1753-6561-3-s7-s99

**Published:** 2009-12-15

**Authors:** Courtney Gray-McGuire, Yeunjoo Song, Nathan J Morris, Catherine M Stein

**Affiliations:** 1 Department of Epidemiology and Biostatistics, Case Western Reserve University, 2103 Cornell Road, Cleveland, Ohio 44106, USA; 2 Oklahoma Medical Research Foundation, 121 North Shartel Avenue, Oklahoma City, Oklahoma 73102, USA

## Abstract

Complex traits are often manifested by multiple correlated traits. One example of this is hypertension (HTN), which is measured on a continuous scale by systolic blood pressure (SBP). Predisposition to HTN is predicted by hyperlipidemia, characterized by elevated triglycerides (TG), low-density lipids (LDL), and high-density lipids (HDL). We hypothesized that the multivariate analysis of TG, LDL, and HDL would be more powerful for detecting HTN genes via linkage analysis compared with univariate analysis of SBP. We conducted linkage analysis of four chromosomal regions known to contain genes associated with HTN using SBP as a measure of HTN in univariate Haseman-Elston regression and using the correlated traits TG, LDL, and HDL in multivariate Haseman-Elston regression. All analyses were conducted using the Framingham Heart Study data. We found that multivariate linkage analysis was better able to detect chromosomal regions in which the angiotensinogen, angiotensin receptor, guanine nucleotide-binding protein 3, and prostaglandin I2 synthase genes reside. Univariate linkage analysis only detected the *AGT *gene. We conclude that multivariate analysis is appropriate for the analysis of multiple correlated phenotypes, and our findings suggest that it may yield new linkage signals undetected by univariate analysis.

## Background

Many common diseases are characterized by several correlated factors. These may be the results of a battery of test scores or they may be series of serum lipid levels or anthropometric measures. It is likely that these correlated traits are influenced by common genes (pleiotropy) or at least genes in common pathways. Eaves et al. [1] point out that the covariance induced on a set of phenotypes segregating at one locus may differ from that induced by segregation at another locus, making the interpretation of univariate results quite difficult [2]. Additionally, the use of multivariate approaches can increase the power and precision of linkage estimates [3,4] and can serve as a mechanism by which to control for multiple comparisons when there are several traits of interest [5].

Hypertension (HTN), defined by consistent, elevated blood pressure (systolic (SBP) and/or diastolic (DBP)) is an example of a multifactorial trait correlated with multiple other phenotypes. Certainly, environmental factors such as diet and exercise are important determinants of HTN, but the influence of genetic factors is also well supported. In fact, there are a small percentage of HTN cases with monogenic forms of the disease [6]. The results of several linkage and association studies of HTN and SBP have suggested candidate genes, including: angiotensinogen (*AGT*) [7], tumor necrosis factor receptor-2 (*TNFR2*) [8], endothelin-converting enzyme-1 (*ECE1*) [9], angiotensin receptor (*AGTR1*), beta-3 subunit of guanine nucleotide-binding protein (*GNB3*) [10], and prostaglandin I2 synthase (*PTGIS*) [11].

This study compares a univariate and multivariate method for linkage analysis using a measure HTN, specifically SBP, and then a set of correlated phenotypes influencing SBP as examples and using the location of established candidate genes as our metric. It is our contention that by using information from multiple factors correlated with SBP levels and each other (rather than either the single continuous or dichotomous trait), we will be more effective in identifying regions of the genome previously demonstrated to be linked to SBP levels without as great a penalty for multiple testing.

## Methods

### Phenotype data

We analyzed the Framingham Heart Study data including observations for Original, Offspring, and Generation 3 cohorts as long as data for all the traits of interest were present. Data were obtained and used in compliance with the data use agreement and Case Western Reserve University Institutional Review Board approval. Low-density lipoprotein (LDL) values were derived using high-density lipoprotein (HDL) and total cholesterol values as required by the Friedewald equation. We used data from the last visit for the Original and Offspring cohorts where all variables of interest were measured. There was only one observation available for the Generation 3 cohort, so that is what we used. The choice of using the latest time point was made in an effort to obtain the most extreme values in our phenotypes of interest (because the study participants would be older).

Based on preliminary model-fitting statistics, we adjusted for age at exam, sex, and the interaction of age at exam by sex by including them as covariates in all analyses. We adjusted for possible HTN treatment by adding a constant of 10 to SBP [12]. Finally, we applied a natural log transformation to triglyceride (TG), HDL, and LDL before analysis to best approximate normality. Within-individual and sibling pair correlations across traits were estimated using FCOR (S.A.G.E. v5.4.1).

### Marker data

Because the purpose of our study is to demonstrate the utility of a multivariate linkage method, using the full set of 500 k genome-wide single-nucleotide polymorphisms (SNPs) would lead to far too much redundancy in the data (due to linkage disequilibrium). Therefore, we selected markers every 1000 kb (i.e., approximately every centimorgan) on which to perform linkage analysis. We further reduced the size of the dataset of analyses by choosing only chromosomes on which there were both previously published linkage signals and candidate genes, including chromosomes 1, 3, 12, and 20, containing candidate genes *AGT *(204 cM), *TNFR2 *(13 cM), and *ECE1 *(21.5 cM); *AGTR1 *(150 cM); *GNB3 *(6.8 cM); and *PTGIS *(47.5 cM), respectively. Our final marker list comprised 611 SNPs.

### Linkage analysis

Prior to linkage analysis, mendelian inconsistencies were identified in the data using MARKERINFO (S.A.G.E. v5.4.1) and the genotypes of all individuals in a family with an inconsistency were set to missing for the given marker. GENIBD was used to estimate the proportion of alleles shared identically by descent (IBD) between sibling pairs using information from individual and neighboring markers (i.e., multipoint). Parental genotypes from the original cohort were used where available in the estimation of IBD sharing. Four pedigrees with complex structure and more than 200 members were removed before IBD sharing estimation.

The univariate phenotype of interest was the quantitative trait SBP, a measure of hypertension. The multivariate traits comprised three phenotypes highly correlated with SBP: TG, HDL, and LDL (Table 1). Thus, we used univariate linkage analysis to analyze SBP and multivariate linkage to analyze TG, HDL, and LDL jointly.

**Table 1:** Within-individual and sibling-sibling pair correlations for TG, HDL, LDL, and SBP^a^

	SBP	TG	HDL	LDL
SBP	**0.2417^b^**	**0.0821^b^**	**-0.0283**	**-0.0292**
TG	0.2726^b^	**0.1987^b^**	**-0.1222^b^**	**0.0440^c^**
HDL	-0.1293^b^	-0.5124^b^	**0.2091^b^**	**0.0153**
LDL	0.1464^b^	0.1684^b^	**-0.1246^b^**	**0.1979^a^**

^a^Within-individual cross-trait correlations are shown in the lower triangle of the table (unbolded font); sibpair cross-trait correlations are in bold font. Correlations bolded on the diagonal are sibpair correlations within trait

^b^Indicates significance at *p *< 0.0001

^c^Indicates significance at *p *< 0.01

#### Univariate linkage

We used performed Haseman-Elston regression [13] on the transformed SBP levels. As implemented in SIBPAL, the Haseman-Elston method regresses a weighted combination of the squared trait difference and squared mean-corrected trait sum on the estimated proportion of alleles shared IBD to account for the non-independence of the sums and differences, as well as the non-independence of sibling-pairs (option W4). Our final sample comprised 3985 full and half-sibling pairs.

#### Multivariate linkage

To conduct multivariate linkage, we used the new S.A.G.E. program RELPAL, which implements a test similar to the multivariate Haseman-Elston [14]. This model is built on the two-level Haseman-Elston [15], which incorporates individual-level covariates at the first level, and performs linkage analysis of multiple traits at the second level. A one-sided score test was used which is asymptotically equivalent to the likelihood-ratio test [16]. Because this method uses a robust sandwich-type estimator, it should maintain correct type I error asymptotically even when the data do not follow a multivariate normal distribution. Significance levels were determined using a novel algorithm described elsewhere [17]. This method has an advantage over other multivariate methods because it retains the power associated with variance-components models while still being robust to normality assumptions. Our final sample comprised 3940 full and half-sibling pairs. The reduction from the 3985 in our univariate analysis was due to the requirement of complete data for all three traits of interest.

## Results

### Correlations

Cross-trait correlations, both within individual and sibling pair, are shown in Table 1. All within-individual cross-trait correlations were significant at *p *< 0.0001 (Table 1), demonstrating two things: first, that TG, HDL, and LDL are indeed adequate surrogates for SBP, and second, that the shared variance between these two traits implies possible pleiotropic effects. Unlike the within-individual correlations, the sibling correlations for SBP with HDL and LDL were not significant. However, TG, HDL, and LDL in one sibling were all significantly correlated with TG, HDL, and LDL in the other sib at *p *< 0.01. The latter result supports the usefulness of joint analysis of these traits (i.e., identification of common genetic determinants), while the former gives credence to the multivariate analysis because the subphenotypes appear to co-vary within a family more than does the univariate trait SBP.

### Linkage analysis

**Figure 1:**
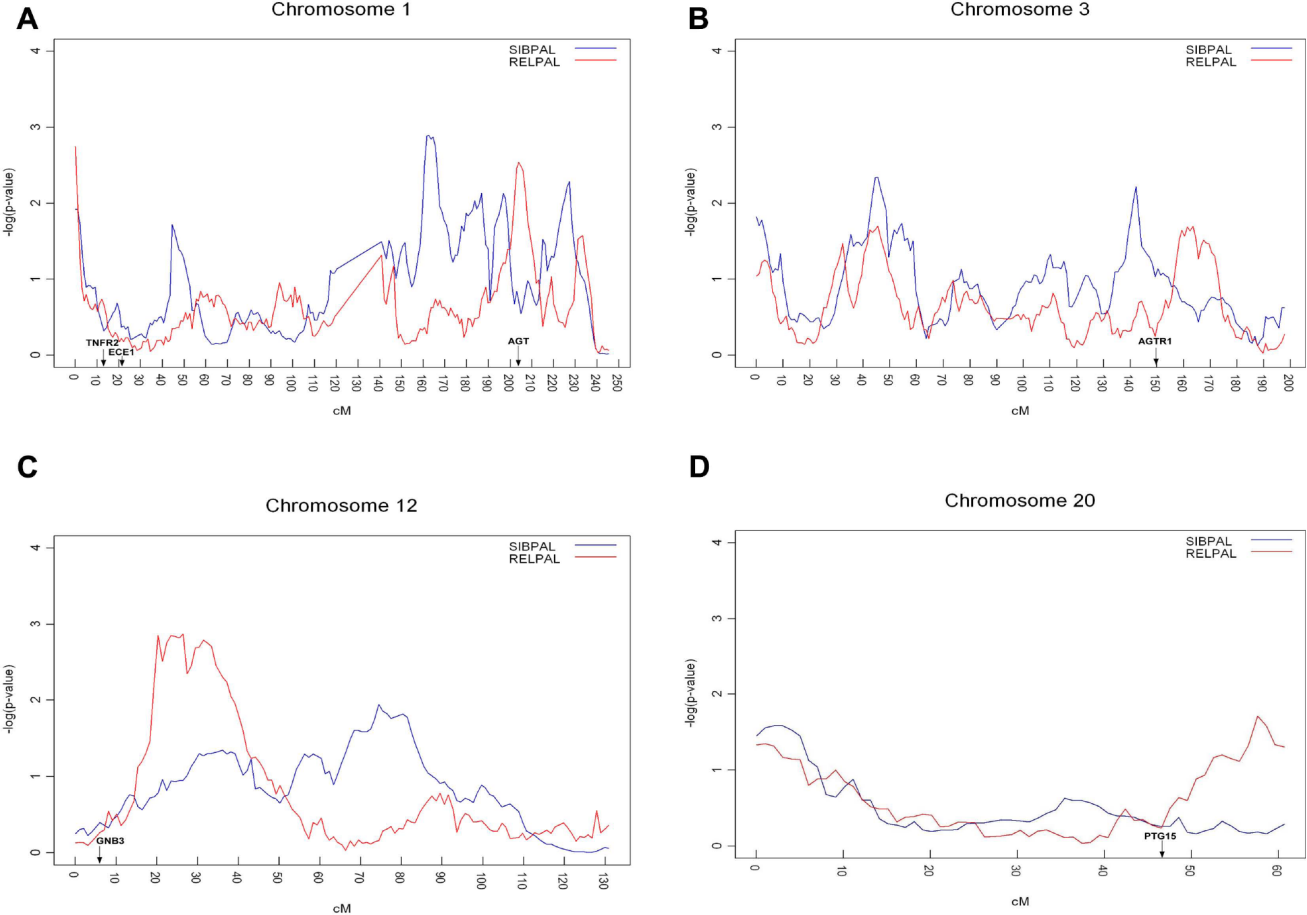
**Univariate and multivariate linkage analysis results for chromosomes 1 (A), 3 (B), 12 (C), and 20 (D)**. -Log_10_(*p*-value) is plotted against marker location in centimorgans. Univariate results are plotted in red, multivariate results are plotted in blue. Locations of relevant candidate genes also indicated.

Of the four chromosomal regions analyzed, we found a few regions of note linked to SBP using the univariate analysis at the α = 0.01 level. These regions were on chromosome 1 between 159 and 172 cM, at 186 cM, and between 195 and 198 cM (Figure 1). These results are within 6 to 32 cM of the *AGT *gene (204 cM) and therefore could be representative of this effect [18], but are certainly not precise enough to rule out the effect of other genes in these regions.

Using the multivariate model, we detected two regions significant at α = 0.01. On chromosome 1 (Figure 1A) we detected a region between 198 and 209 cM and also at the first SNP. The *AGT *gene is contained within this first interval (204 cM) and *TNFR2 *is located at 13 cM, very near to the first SNP analyzed. On chromosome 12 (Figure 1C), we observed linkage between 18 and 35 cM; again, within 9 cM of the previously associated *GNB *gene. At the α = 0.05 level, we observed linkage on chromosome 3 (Figure 1B) between 158 and 171 cM, 8 cM from *AGTR1*.

## Discussion

In this study, our objective was to compare univariate and multivariate linkage results of four chromosomal regions known to contain mendelian genes linked to HTN. Linkage analysis remains a relevant approach for the analysis of rare and/or mendelian genetic effects [19,20], as well as for providing *a priori *weights for association analysis [21], so we examined a newly implemented and truly multivariate linkage analysis model. We recognize that the most compelling demonstration of new methodology is via simulated data. However, the effect sizes represented in the real data were much more suited to linkage analysis. And, because there were established effects to which we could compare our results, this data represented a reasonable alternative. Indeed, we used a univariate and multivariate linkage approach to analyze SBP or traits related to HTN (and highly correlated with SBP) - TG, LDL, and HDL, respectively. We examined the same regions known to contain genes predisposing to risk of HTN. Our multivariate linkage analysis identified more nominally significant regions, and these results covered the chromosomal regions where the *AGT*, *TNFR2*, *AGTR*, and *GNB3 *genes reside. Though the univariate results were near the AGT gene, the multivariate results identified this genomic region more precisely. There were also univariate linkage findings in the vicinity of *GNB3 *and *PTGIS*, but not nearly as significant as the multivariate findings (Figures 1, C and 1D). These results demonstrate the usefulness of multivariate linkage analysis in mapping complex traits such as HTN, particularly those for which there are highly correlated subphenotypes with large within-family covariance.

## Conclusion

In summary, we observed linkage to chromosomal regions containing candidate genes for HTN. Our multivariate analysis identified more such regions than our univariate analysis. These findings support the use of multivariate linkage analysis when analyzing a number of correlated phenotypes that together predispose to a complex trait like HTN.

## List of abbreviations used

DBP: Diastolic blood pressure; HDL: High-density lipoprotein; HTN: Hypertension; IBD: Identical by descent; LDL: Low-density lipoprotein; SBP: Systolic blood pressure; SNP: Single-nucleotide polymorphism; TG: Triglyceride

## Competing interests

The authors declare that they have no competing interests.

## Authors' contributions

CG-M and CMS conceived of and designed the study and drafted the manuscript. NJM helped conduct the study and draft the manuscript. YS performed the statistical analysis. All authors read and approved the final manuscript.
